# Bilateral Thalamic Stroke as a Cause of Decreased Responsiveness

**DOI:** 10.7759/cureus.14935

**Published:** 2021-05-10

**Authors:** Mansoor Qureshi, Marvi Qureshi, Muhammad Gul, David Lebowitz, Latha Ganti

**Affiliations:** 1 Biomedical Sciences, University of Central Florida, Orlando, USA; 2 Emergency Medicine, HCA Healthcare Graduate Medical Education Consortium Emergency Medicine Residency Program of Greater Orlando, University of Central Florida, Orlando, USA; 3 Emergency Medicine, Osceola Regional Medical Center, Kissimmee, USA; 4 Internal Medicine, Orlando Health Orlando Regional Medical Center, Orlando, USA; 5 Emergency Medicine, University of Central Florida College of Medicine, Orlando, USA; 6 Emergency Medicine, Envision Physician Services, Plantation, USA

**Keywords:** acute stroke, thalamic infarct, ischemic stroke, cerebrovascular disease, emergency department

## Abstract

We report the case of a 77-year-old male with no prior history of stroke who came in as a stroke alert for right facial droop and speech slurring, but upon presentation he had decreased responsiveness. Initial imaging for stroke was negative. Laboratory evaluation revealed no abnormalities. As lumbar puncture was about to be performed, the patient had a sudden resolution of symptoms, became responsive, and started answering questions. Magnetic resonance imaging (MRI) revealed small acute infarcts in the bilateral thalami and adjacent central aspect of the midbrain, right larger than the left. General decreased responsiveness needs to be considered in the differential diagnosis of stroke.

## Introduction

Acute stroke is a medical emergency with potential interventions that are time-sensitive; thus, prompt recognition is of paramount importance. The first recognition needs to occur by the patient or the family for them to get medical attention in the first place. Indeed, even when patients think they may be having a stroke, they do not always present promptly, and even less so when they are unsure [1]. The next recognition needs to occur by the emergency medical services (EMS) personnel and the emergency physician. Most are aware of focal weakness and speech deficits as being signs of acute stroke. Atypical symptoms, however, is where there is great potential to miss the diagnosis of stroke [2].

We present the case of a 77-year-old male with no prior history of stroke and myocardial infarction, not on anticoagulation, who presented primarily with decreased responsiveness with negative initial brain imaging, but later he was found to have bilateral thalami ischemic stroke on magnetic resonance imaging (MRI).

## Case presentation

A 77-year-old male with a past medical history significant only for gout, and hypertension presented as a stroke alert. His last known normal time was 12 hours prior, before going to sleep. His wife noted an episode of right facial droop and speech slurring, which prompted her to call emergency medical services. His wife also described an episode of thrashing in the bed and urination. She mentioned that the day before presentation the patient was “not feeling well” and took aspirin. He did not mention experiencing any specific symptoms.

**Figure 1:**
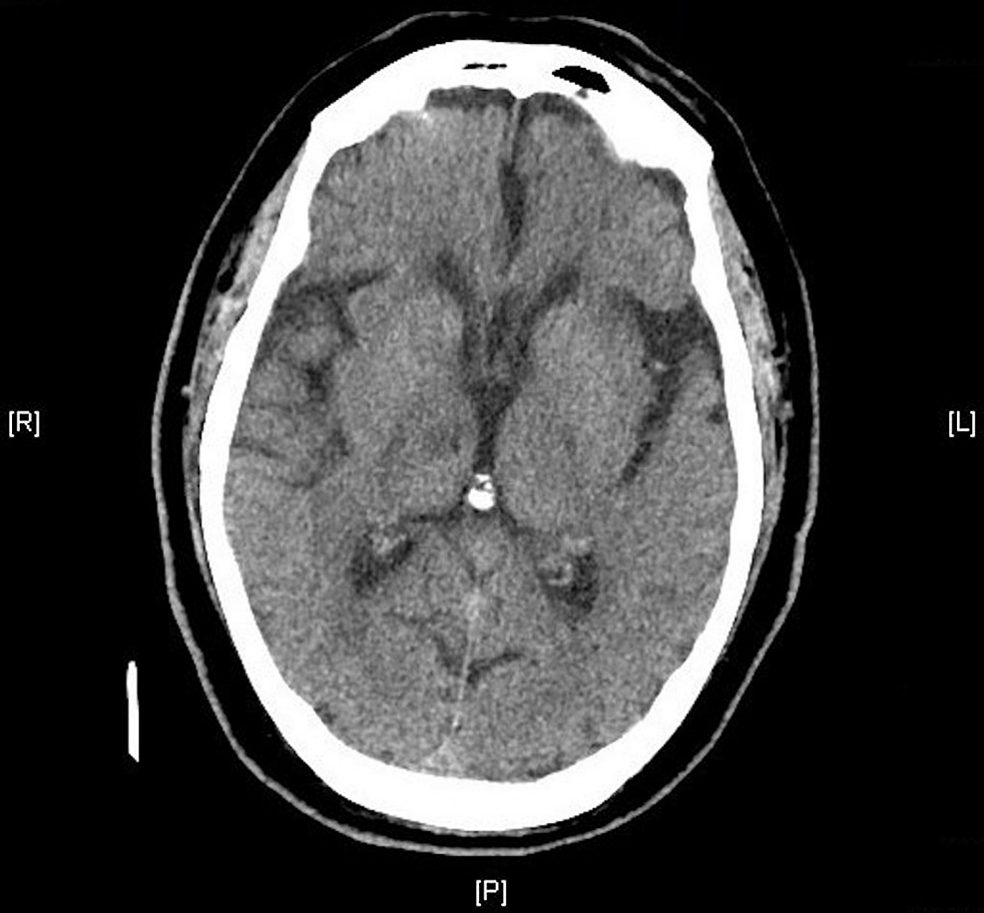
Initial “stroke alert” CT, which did not reveal any evidence of hemorrhage or infarct. CT, computed tomography

Upon presentation to the emergency department, the patient could localize to pain but had a decreased level of consciousness. Vital signs were unremarkable, but frequent premature ventricular complexes were seen on the electrocardiogram. The patient’s wife did not know of any prior history of this. Physical examination was remarkable for 5-mm dilated pupils bilaterally, crackles bilaterally at the lung bases, and decreased level of consciousness. The National Institutes of Health Stroke Score (NIHSS) was 18: 2 for level of consciousness, 3 for visual fields, 2 for the left arm, 2 for the right arm, 2 for the left leg, 2 for the right leg, 3 for best language, and 2 for dysarthria. Laboratory evaluation revealed no leukocytosis, a normal glucose of 115 mg/dL, and lactic acid of 1.4 mmol/L. Urine drug screen was negative, as was the urinalysis. The patient had normal vital signs and thus did not meet the criteria for sepsis. Initial computed tomography (CT) of the brain did not reveal an infarct or hemorrhage (Figure 1).

CT angiography (CTA) of the head and neck did not show any sign of aneurysm but revealed scattered carotid calcifications, a left parotid mass of 2 cm, and a right apical cavitary lesion with peripheral calcifications confirmed by chest radiography (CXR). CXR also revealed several small pulmonary nodules scattered in both lungs as well as a widened upper mediastinum. The patient was still not responsive after imaging was performed, and a lumbar puncture was about to be performed since the patient still had altered mental status. As consent was obtained, the patient slowly became responsive, and started answering questions and opening his eyes. Due to acute improvement in mental status, lumbar puncture was not performed.

**Figure 2:**
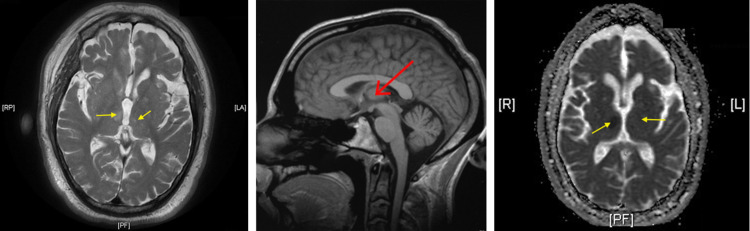
MRI demonstrating right greater than left bilateral thalamic infarcts (yellow arrows). The left panel is the axial T2 FLAIR image, the center panel shows the infarct on sagittal view (red arrow), and the right panel is the ADC image. MRI, magnetic resonance imaging; FLAIR, fluid-attenuated image recovery; ADC, apparent diffusion coefficient

MRI was then performed, which revealed a small acute infarct in the bilateral thalami and adjacent central aspect of the midbrain, right larger than the left (Figure 2). The patient was admitted for further neurological workup.

## Discussion

We reported a case of a patient who presented with primary complaint of altered mental status who had initial imaging of CT and CTA negative for stroke. The patient acutely experienced improvement in symptoms as a lumbar puncture was being planned to determine the cause of altered mental status. Further workup including MRI revealed bilateral thalami infarcts.

The thalamus is a structure that relays motor and sensory information to the cerebral cortex. Damage to the anterior nucleus of the thalamus can produce neuropsychological disturbances [3]. Early involvement can manifest as decreased level of consciousness that can last from hours to days, including features such as confusion, agitation, and aggression [4,5]. As such, it is important to consider decreased responsiveness as a potential thalamic stroke symptom and keep ischemic stroke on the differential.

Thalamic infarcts can present in various ways depending on the specific location within the thalamus and the extent of the infarct within it. Lateral thalamic infarcts often present with pure sensory deficits [6]. When they do involve sensorimotor deficits, the deficits are ipsilateral and spare consciousness. Paramedian thalamic infarcts usually lead a patient to be initially lethargic and difficult to arouse, but then emerge into alertness [6], as did our patient.

Many atypical presentations of strokes have been reported, including a foreign body sensation in the oropharynx [7], thrombotic thrombocytopenic purpura, and giant cell arteritis [8], to name a few.

The initial imaging performed for our patient, including CT and CTA of the head, was negative for stroke. It was only after an MRI was performed did we discover the bilateral thalami stroke. As such, it is important to consider stroke even in a patient such as ours, who was correctly stroke alerted by EMS but had negative initial imaging.

## Conclusions

Stroke should be considered as a potential diagnosis in a patient presenting with altered mental status. This is especially important considering the time-sensitive nature of potential interventions for acute ischemic stroke.
